# Physical inactivity is strongly associated with anxiety and depression in Iraqi immigrants to Sweden: a cross-sectional study

**DOI:** 10.1186/1471-2458-14-502

**Published:** 2014-05-25

**Authors:** Faiza Siddiqui, Ulf Lindblad, Louise Bennet

**Affiliations:** 1 Center for Primary Health Care Research, Region Skåne and Lund University, Malmo, Sweden; 2 Department of Primary Health Care, Sahlgrenska Academy, University of Gothenburg, Gothenburg, Sweden; 3 Department of Clinical Sciences, Lund University, Skåne University Hospital, Malmö, Sweden; 4 Genetic and Molecular Epidemiology, Lund University, Malmö, Sweden; 5 Center for Primary Health Care Research, Clinical Research Centre, Lund University, Skåne University Hospital, Building 60, Floor 12, Jan Waldenströms gata 37, 205 02 Malmö, Sweden

**Keywords:** Anxiety, Depression, Physical activity, Immigrants, Middle East, Sweden

## Abstract

**Background:**

Increasing evidence on associations between mental health and chronic diseases like cardio-vascular disease and diabetes together with the fact that little is known about the prevalence of anxiety/depression and associated risk factors among Iraqi immigrants to Sweden, warrants a study in this group. The aim was to study the prevalence of anxiety and depression in immigrants from Iraq compared to native Swedes and compare socioeconomic and lifestyle-related factors associated with these conditions.

**Method:**

A population-based, cross-sectional study of residents of Malmö, Sweden, aged 30–75 years, born in Iraq or Sweden. The overall response rate was 49% for Iraqis and 32% for Swedes. Anxiety and depression were assessed using the Hospital Anxiety and Depression Scale. Associations were studied using multivariate logistic regression models. The outcome was odds of depression and/or anxiety.

**Results:**

Compared to Swedes (n = 634), anxiety was three times as prevalent (52.6 vs. 16.3%, *p* < 0.001) and depression five times as prevalent (16.3 vs. 3.1%, *p* < 0.001) in Iraqi immigrants (n = 1255). Iraqis were three times more likely to be anxious and/or depressed compared to Swedes (odds ratio (OR) 3.02, 95% confidence interval (CI) 2.06-4.41). Among Iraqis, physical inactivity (<150 min/week) (OR 2.00, 95% CI 1.49-2.69), economic insecurity (OR 2.16, 95% CI 1.56-3.01), inability to trust people (OR 1.75, 95% CI 1.28-2.39) and smoking (OR 1.43, 95% CI 1.02-2.01), were strongly associated with anxiety/depression. Among Swedes, living alone (OR 2.10, 95% CI 1.36-3.25) and economic insecurity (OR 2.38, 95% CI 1.38-4.12) showed the strongest associations with anxiety/depression. Country of birth modified the effect of physical inactivity (*P*_*interaction*_ =0.058) as well as of marital status (*P*_*interaction*_ =0.001).

**Conclusion:**

Our study indicates that economic insecurity has a major impact on poor mental health irrespective of ethnic background but that physical inactivity may be more strongly associated with anxiety/depression in immigrants from the Middle East compared to native Swedes. Preventive actions emphasizing increased physical activity may reduce the risk of poor mental health in immigrants from the Middle East, however intervention studies are warranted to test this hypothesis.

## Background

Political instability over the past few decades in different parts of the world has resulted in a rapid increase in the number of immigrants. International immigrants account for 3.1% of world’s total population and 8.7% of the European population [1]. Iraqi immigrants constitute the largest non-European immigrant group in Sweden. According to the statistics bureau Statistics Sweden, the total number of Iraqi immigrants in Sweden in 2012 was 127,860, with most of them residing in the municipalities of Malmö and Södertälje [2].

Mental ill-health in Sweden has been on rise since 1990s with consequent effects on economy and productivity. Studies have reported increasing levels of psychological distress, anxiety and suicidal tendencies especially among young Swedes [3]. Poor mental health is considered as the major cause of labor market exclusion in young people as well as disability pensions and health care spending in older age groups in Sweden [4]. Non-European immigrants in Sweden are considered to be at particularly higher risk of mental health problems because of increased exposure to risk factors like poor social support, unemployment and financial instability [5]. Apart from their adverse effects on quality of life, mental health conditions like anxiety and depression are also associated with chronic diseases such as cardiovascular diseases, hypertension and diabetes [6-9]. Depressive symptoms are also associated with poor metabolic control in diabetes due to failure to adhere to self-care behaviors relating to diet, exercise and medication [10]. According to World Health Organization (WHO) projections, by 2030, heart diseases and depression will be the two leading causes of loss of disability-adjusted life years in high-income countries [11]. In spite of the fact that Iraqi immigrants represent a large proportion of Swedish residents, research on the mental health of Iraqi immigrants in Sweden is sparse. Two studies on Middle Eastern immigrants have shown a higher prevalence of mental ill-health in this group and its association with gender, economic insecurity, poor social network and poor socio-cultural adaptation [12,13]. It is important to study the mental health of Iraqi immigrants in Sweden in order to improve the prevention of chronic diseases, as well as to improve the provision of mental health services and quality of life in this group.

The primary aim of this survey was to study the prevalence of anxiety and depression among residents of Malmö born in Iraq and to compare it with that in residents born in Sweden. A secondary objective was to identify socioeconomic and lifestyle-associated risk factors with anxiety and depression and to determine whether these risk factors differ between Iraqi and Swedish participants.

## Methods

**Figure 1:**
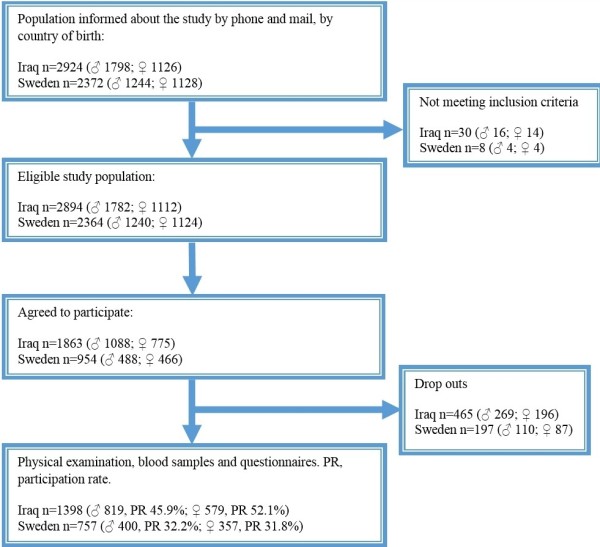
Flow diagram of the recruitment of the study population.

This study is based on data gathered during the MEDIM study, which was conducted in Malmö, Sweden between February 1, 2010 and December 31, 2012. Malmö is a multicultural city and according to 2011 estimates 32% of its residents were born abroad. Iraqis constitute the second largest immigrant group in Sweden and the largest immigrant group in Malmö, with a population of 9000 among its 300,000 inhabitants. Citizens of Malmö born in Iraq or Sweden were randomly selected from a census register and invited by mail to participate in a population-based survey that included a physical examination, questionnaires, blood tests and assessment of lifestyle habits. A flow diagram outlining the recruitment of the study population is presented in Figure 1. The overall response rate was 49% for Iraqis and 32% for Swedes.

The outcome variable was anxiety and/or depression and was measured using the Hospital Anxiety and Depression Scale (HADS), which is a validated and reliable measure for screening anxiety and depression among patients in general medical clinics [14]. Individuals are asked to respond to 14 questions, seven relating to anxiety and seven to depression. For each question there are four possible responses that are scored from 0 to 3 and are summed to obtain separate anxiety and depression scores. A score of 0–7 indicates no anxiety or depression; 8–10 indicates a doubtful case whereas 11–21 indicates moderate to severe anxiety or depression [15]. All study participants with anxiety or depression scores of 11–21 and/or receiving anti-depressants, which was self-reported by participants, were classified as anxious and/or depressed whereas the rest were classified as non-anxious and/or non-depressed.

*Age* was calculated from the date of birth until the date of examination. *Marital status* was dichotomized as married/living with a partner and living alone. *Height* was measured to the nearest centimeter using a wall-mounted stadiometer. *Weight* was measured to the nearest kilogram with participants wearing light weight clothes and without shoes using a calibrated electronic scale. *Body Mass Index* (BMI) was calculated by dividing weight (kg) by height squared (m^2^). *Physical inactivity* was defined as less than 150 min of physical activity per week [16]. *Education* was classified in two groups: less than high school and high school or more. Participants were considered *unemployed* if they did not have work for the last 12 months. *Economic insecurity* was defined as inability to secure 15000 SEK in one week in an unexpected/unforeseen situation. Participants were considered *non-smokers* if they had never smoked or stopped smoking more than six months ago. *Alcohol consumption* was assessed using the question “Do you drink alcohol?”

Two social capital-related variables, *participation in social activities* and *mutual trust*, were included. Participation in social activities was assessed using 13 questions asking participants whether they had participated in any of the following activities: study group at the work place, study group in spare time, union meeting, society meeting, theatre/cinema, art exhibition, church, sport, letter to newspapers, demonstration, night club/dance party, large family gathering and private party at somebody’s home. Responses to these questions were summed to obtain the number of social activities participated in the last 12 months (0–13). Participation in fewer than four social activities in last 12 months was regarded as low social participation. A missing response to any one of the questions was interpreted as a negative response, but if the responses to all questions were missing, the participant was excluded. *Mutual trust* was assessed using the question “Do you think that you can trust most people in general?” (yes/no).

Acculturation to Swedish society among Iraqi participants was assessed using two separate questions about Swedish *reading* and *writing skills*. Individuals who always or almost always read and wrote in Swedish were considered better acculturated than those who never, almost never or sometimes read and wrote in Swedish. *Time since migration* was calculated from year of migration and was included as a continuous variable.

Statistical analysis was performed using SPSS 21.0 for Windows XP. The prevalence of potential confounding factors was compared between non-anxious/non-depressed and anxious/depressed participants in Iraqis and Swedes. Differences in means between groups were adjusted for age and sex using general linear models (Table 1). Differences in proportions between groups were adjusted for age and sex using binary logistic regression models when appropriate (Table 1). Associations between anxiety/depression and socioeconomic, lifestyle and mental health-related risk factors were estimated using multivariate logistic regression for both Iraqis and Swedes (Table 2). In Iraqis, additional analysis was done including acculturation-related variables (represented by time since migration, never read in Swedish and never write in Swedish). Associations are expressed as odds ratios (ORs) with 95% confidence intervals (CIs). All tests were two-sided and a *p*-value of < 0.05 was considered statistically significant.

**Table 1:** Characteristics of the study participants

**Variable**	**Born in Iraq N = 1255**			**Born in Sweden N = 634**		
	**Not anxious or depressed**	**Anxious and/or depressed**	***p***	**Not anxious or depressed**	**Anxious and/or depressed**	***p***
	**N = 383**	**N = 872**		**N = 488**	**N = 146**	
	**(30.5%)**	**(69.5%)**		**(77.0%)**	**(23.0%)**	
Mean age (years)	46.5 (10.3)	45.1(9.0)	0.014	48.9 (11.2)	46 (10.3)	0.007
Male sex, n (%)	267 (69.7)	479 (54.9)	< 0.001	269 (55.1)	65 (44.5)	0.025
Living alone, n (%)	32 (8.9)	60 (7.7)	0.733	108 (23.9)	54 (42.2)	< 0.001
Mean BMI (kg/m^2^)	28.9 (4.2)	29.4 (4.6)	0.073	27.2 (4.6)	26.9 (5.0)	0.777
Physical activity < 150 min/week, n (%)	230 (62.2)	661 (78.5)	< 0.001	152 (31.1)	57 (39.0)	0.054
Education level < HS, n (%)	82 (21.4)	263 (30.2)	0.014	78 (16.0)	18 (12.3)	0.754
Unemployed, n (%)	39 (10.2)	90 (10.3)	0.786	11 (2.3)	6 (4.1)	0.316
Economic insecurity, n (%)	265 (69.2)	738 (84.6)	< 0.001	62 (12.7)	48 (32.9)	< 0.001
Can’t trust people, n (%)	277 (72.3)	698 (80.1)	0.003	95 (19.5)	40 (27.4)	0.069
Low social participation*, n (%)	306 (79.9)	760 (87.2)	0.004	158 (32.4)	70 (47.9)	< 0.001
Mean time since migration, (years)	20.1 (10.0)	17.5 (9.3)	0.001	-	-	-
Never read in Swedish, n (%)	239 (62.4)	635 (72.8)	< 0.001	-	-	-
Never write in Swedish, n (%)	228 (59.5)	584 (67.0)	0.002	-	-	-
Smoking, n (%)	81 (21.2)	215 (24.7)	0.016	112 (23.0)	37 (25.3)	0.641
Alcohol consumption, n (%)	88 (23.0)	151 (17.5)	0.491	425 (87.3)	112 (76.7)	0.005

BMI = body mass index, HS = high school.

*Fewer than four social activities in the last 12 months.

**Table 2:** Associations between anxiety/depression and socioeconomic risk factors, in participants born in Iraq and Sweden

**Risk factor**	**Born in Iraq**			**Born in Sweden**		
	**N = 1095**			**N = 577**		
	**OR**	**95% CI**		**OR**	**95% CI**	
Age (years)	0.99	0.97	1.00	0.98^+^	0.95	0.99
Male sex	0.69^+^	0.51	0.94	0.76	0.50	1.16
Living alone	0.73	0.45	1.19	2.10^++^	1.36	3.25
BMI (kg/m^2^)	1.02	0.99	1.06	0.98	0.94	1.03
Physical activity < 150 min/week	2.00^+++^	1.49	2.69	1.47	0.95	2.29
Education level < HS	1.28	0.93	1.77	0.72	0.35	1.48
Unemployed	1.11	0.71	1.73	0.64	0.17	2.37
Economic insecurity	2.16^+++^	1.56	3.01	2.38^++^	1.38	4.12
Can’t trust people	1.75^+++^	1.28	2.39	1.07	0.64	1.77
Low social participation*	1.08	0.75	1.57	1.51	0.94	2.43
Smoking	1.43^+^	1.02	2.01	0.85	0.51	1.40
Alcohol consumption	0.94	0.66	1.34	0.82	0.45	1.48

BMI = body mass index, HS = high school.

Data were adjusted for age, male sex, living alone, BMI, physical activity less than 150 min/week, education less than high school, unemployment, economic insecurity, trust in people, social participation, smoking and alcohol consumption.

*Participation in fewer than four social activities in last 12 months.

Associations are expressed as odds ratios (OR) with 95% confidence intervals (CIs) and were calculated using logistic regression.

^+^p-value < 0.05, ^++^p-value < 0.01, ^+++^p-value < 0.001.

Interactions were tested between country of birth and other independent risk factors for anxiety/depression. Multicollinearity was tested for but was not considered an issue since all variance inflation factors (VIFs) in the multivariate regression models had values of < 2.0.

### Ethical considerations

This study is compliant to ethical guidelines provided in Helsinki Declaration. The research was approved by Ethics committee at Lund University (No. 2009/36 & 2010/561) and written informed consent was obtained from all participants.

## Results

**Figure 2:**
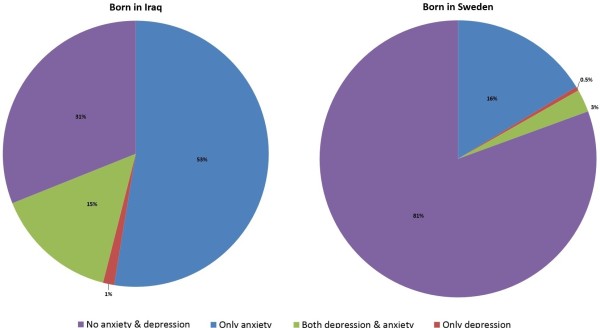
Prevalence of anxiety and/or depression in participants born in Iraq (left) or Sweden (right).

Information on anxiety and depression was available for 1889 of the 2155 study participants. Of these participants, 872 Iraqis (69.5%) and 146 Swedes (23.0%) were found to be anxious and/or depressed and/or were taking anti-depressants. The prevalence of ongoing moderate to severe anxiety (52.6 vs. 16.3%, *p*-value < 0.001) and moderate to severe depression (16.3 vs. 3.1%, p-value < 0.001), as defined by the HADS, was considerably higher among Iraqi immigrants compared to native Swedes as shown in Figure 2. Anxious and/or depressed Iraqis were younger, more physically inactive and more socioeconomically disadvantaged than their fellow countrymen in the non-anxious non-depressed group; however, there were no differences in BMI, marital status and employment status. There were significant differences between the two subgroups in time since migration and Swedish reading and writing skills, as shown in Table 1. In Swedish participants, there were differences between the anxious and/or depressed and non-anxious non-depressed subgroups in terms of age, sex, marital status, physical activity, economic security, alcohol consumption and social participation.

In the regression analysis, Iraqis had three times higher odds of anxiety and depression (OR 3.02, 95% CI 2.06-4.41) compared to Swedes, after adjustment for age, sex, marital status, economic insecurity, education, employment status, physical activity, BMI, smoking, alcohol consumption, mutual trust and social participation. Among Iraqi-born participants, physical inactivity and economic insecurity contributed the most to increased odds of anxiety and depression, followed by smoking and mutual trust. There was an interaction within the level of significance between country of birth and physical activity <150 min/week (*P*_*interaction*_ =0.058). Among native Swedes economic insecurity had the strongest association with anxiety and depression, followed by marital status and younger age, as shown in Table 2. The fact that marital status was associated with depression and/or anxiety in Swedes only was confirmed by an interaction between country of birth and marital status (*P*_*interaction*_ =0.001).

In Iraqis, additional analysis was done to adjust for acculturation status represented by time since migration, never read in Swedish and never write in Swedish. However these variables were not significant and didn’t effect the existing associations.

## Discussion

This study reveals a three times higher prevalence of anxiety and a five times higher prevalence of depression among Iraqi immigrants compared to native Swedes in the study population. Moreover, even after adjusting for potential confounders, Iraqis had three times higher odds of moderate to severe anxiety and/or depression compared to Swedes. A key finding of this study was that anxiety/depression was strongly associated with physical inactivity in Iraqi immigrants to Sweden, but not in native Swedes. Economic insecurity showed a strong association with anxiety/depression in both Iraqis and Swedes.

Although other studies have reported comparable figures for the prevalence and risk of anxiety and/or depression among non-European immigrants in Sweden, very few have focused solely on Iraqi immigrants. Studies on mental health and ethnicity conducted in Sweden have mainly focused on more severe mental illnesses such as psychosis, suicidal tendency and schizophrenia, as well as intake of psychotropic drugs and refugee immigrants [17,18]. In one study, Tinghög et al., using the Hopkins Symptom Check List 25, demonstrated an anxiety/depression prevalence of 60.6% among Iraqi immigrants in Sweden compared to 11.7% in Finnish immigrants [5]. In this study, female sex, poor social network and economic insecurity were found to be associated with low subjective well-being and anxiety/depression, in line with our findings; however, physical activity was not considered [12]. In another study, Turkish immigrants were found to have two times increased risk for self- reported anxiety than Swedes after adjusting for age and socio-economic status which is comparable to our study as Turkish and Iraqi immigrants exhibit geographical and cultural similarities. The finding that Iraqis have higher odds of anxiety/depression even after adjusting for potential risk factors in the model is in agreement with previous studies in Sweden. These studies indicate that the risk of depression associated with immigrant status cannot be fully explained by socio-economic factors as well as report ethnicity as a risk factor for mental ill-health [5,19,20]. However this finding also indicates the need to further explore and identify variables that are associated with mental health among Iraqis.

The prevalence of anxiety/depression in Swedes was lower as compared to previous studies which reveal an anxiety and depression prevalence of 32% and 7-10% respectively [5,21]. The prevalence estimates, therefore need to be interpreted with care, based on low response rate in Swedes which can result in underestimation. The association of marital status with anxiety/depression in Swedes seen in this study is in line with previous research [5,21].

In many studies, physical activity has been shown to have a mental health-promoting effect in the general population. Clinical trials have revealed that its effects as a therapy for anxiety and depression are comparable to those of anti-depressant medication and psychotherapy [22]; however, the association between physical activity and mental health among immigrants is less frequently discussed compared to general populations and requires further research. In a randomized controlled trial conducted in the Netherlands, a physical activity intervention led to improved mental health among elderly Turkish immigrants [23]. This indirectly supports the finding in the current study that Iraqi immigrants who performed less than 150 min/week of physical activity were more likely to be anxious and/or depressed than their counterparts born in Sweden, further supported by our finding that country of birth modified the effect of physical activity. Moreover, physical activity is supposed to increase norepinephrine neurotransmission in the central nervous system, serotonin synthesis and secretion of atrial natriuretic peptide, all of which are plausible biological explanations for its association with anxiety and depression [24].

In Iraqi immigrants as well as amongst native Swedes, economic insecurity was as strongly associated with anxiety/depression as physical inactivity was. Economic insecurity represents one aspect of poor socioeconomic status, a much discussed factor in the etiology of anxiety and depression [25]. Most studies on the mental health of immigrants take into account employment status, income and education as indicators of socioeconomic status [17,26], but few have also considered economic insecurity [5,12]. The negative association between economic insecurity and the mental health of immigrants observed in these studies, as well as in our study, indicates the need for further research to strengthen existing evidence.

Mutual trust, a component of social capital, turned out to be associated with anxiety and depression in Iraqis. Over the past few years, the concept of social capital has evolved in health promotion research. Mutual trust and social participation, measured as membership of voluntary organizations, have been identified as two core aspects of social capital by many researchers [27]. The association between cognitive components of social capital, i.e., mutual trust, and mental health has also been reported in other studies [28,29]. A bi-directional association between social capital and mental health, according to which poor social capital could be the result or the cause of depressive symptoms, has been proposed. However such an association is more likely between social participation and common mental disorders than between mutual trust and common mental disorders [28].

Time since migration, Swedish reading skills and Swedish writing skills had no influence on anxiety and depression scores in Iraqi immigrants in the multivariate model. This indicates that among Iraqi immigrants in Sweden, who on average had lived in Sweden for 20 years, socioeconomic and lifestyle-related factors such as economic insecurity, feeling of trust in people and physical activity were more important determinants of mental health compared to lack of acculturation. That acculturation has a weak association with mental health in immigrants was also found in a large study of the mental health of aging immigrants in 11 European countries. In this study, Ladin et al. elaborated that acculturation-related variables such as time since migration and citizenship status, both of which are strongly correlated with language skills, were not predictive of depression risk among immigrants [30].

### Strengths & limitations

The current study adds to the existing knowledge on mental health among immigrants in Sweden by providing a comparison of Swedes with Iraqis, the largest non-European immigrant group in Sweden. In addition, while most previous studies measured depression using one or another scale, anxiety was either ignored, replaced by measures of subjective well-being or self-reported. By contrast, the present study used a well validated instrument for detecting depression and anxiety in Iraqi immigrants in Malmö, a group that is at risk of the consequences of poor mental health in the form of chronic diseases and impaired quality of life.

It is also worth mentioning that immigrants tend to have somatic complaints like body pains and other physical symptoms as an expression of underlying psychological illness [13].

The HAD questionnaire used in this study aims to identify anxiety and depression in non-psychiatric clinical settings and tends to rule out somatic symptoms that might originate from physical illness rather than poor mental health. Although Swedish Council on Health Technology Assessment has criticized HADS for its indefinable sensitivity, in a literature review conducted by Bjelland et al., the HADS was found to have good sensitivity and specificity and performed well as an instrument for measuring depression and anxiety caseness [14,31]. These findings were confirmed in a Swedish sample [32]. Moreover in the current study, a large group was found to be anxious/depressed according to HADS which diminishes concerns regarding its sensitivity.

In the multivariate logistic analysis, depression and anxiety were considered together not only because anxiety is highly prevalent amongst patients with depression, but also because it can precede depression in many cases. In addition, shared pharmacotherapy and shared genetic risk indicate a common biological pathway underlying anxiety and depression, which in turn provides grounds for considering them together rather than as separate entities [33,34].

The cross-sectional approach used in the study does not allow a causal association between physical inactivity and mental health among Iraqi immigrants to be established. Physical inactivity could be a consequence of depression rather than a cause as depressed individuals often lack motivation to engage in physical activities [30]. However, considering our findings in relation to results from clinical trials on physical activity and mental health [23,35], it can be inferred that physical activity interventions in clinical settings might prove to be beneficial for anxious and depressed Iraqi immigrants but intervention studies are warranted to prove this relationship.

The association between physical activity and anxiety/depression was near the level of significance for Swedes, but not significant, which we assume is a consequence of the smaller sample size.

The response rates in general were low though in Iraqis, it was comparable to other studies on Iraqi immigrants in Sweden [12]. Nevertheless it should be acknowledged that low response rate can result in underestimation of prevalence rates especially when dealing with mental ill-health [12]. It is alarming that prevalence of anxiety/depression could be even higher than what is reported in this study in both Swedes and Iraqis.

## Conclusion

Iraqi immigrants to Sweden are at increased risk of anxiety and depression compared to native Swedes. Our data indicate that physical inactivity and economic insecurity exhibit strong associations with anxiety and depression among Iraqi immigrants. Preventive actions emphasizing increased physical activity may have a high impact on the risk of poor mental health in this group.

## Abbreviations

BMI: Body mass index; CI: Confidence interval; HADS: Hospital anxiety and depression scale; OR: Odds ratio; WHO: World Health Organization.

## Competing interests

The authors declare that they have no competing interests.

## Authors’ contributions

FS performed the statistical analysis, interpreted the data and wrote the manuscript. UL contributed to the design of the study, interpretation of the data, discussions and writing the manuscript. LB designed the study, collected the data and made it available for analysis. She also assisted with data analysis and interpretation, discussions and writing the manuscript. All authors revised/edited the article critically and have approved the final version of the manuscript.

## Pre-publication history

The pre-publication history for this paper can be accessed here:

http://www.biomedcentral.com/1471-2458/14/502/prepub
